# Measurement of the Vertical Spatial Metaphor of Power Concepts Using the Implicit Relational Assessment Procedure

**DOI:** 10.3389/fpsyg.2019.01422

**Published:** 2019-06-27

**Authors:** Bao Hong, Lu Zhang, Hongri Sun

**Affiliations:** School of Public Administration, Nanchang University, Nanchang, China

**Keywords:** power, spatial metaphor, embodied cognition, relational frame theory, implicit relational assessment procedure

## Abstract

This experimental study explored the use of the implicit relational assessment procedure (IRAP) for understanding the vertical spatial metaphor of power. In the classic IRAP procedure, we formed four sets of stimuli based on the relationship between power words (powerful and powerless words) and vertical position on a computer screen (upper or lower) that were either pro-metaphor (i.e., powerful-high, powerless-low) or anti-metaphor (i.e., powerful-low, powerless-high). Participants were then asked to judge whether the words were consistent or inconsistent with the set of instructions given to them. We found that the *D*_irap_ scores of powerful words in an above vertical space and powerless words in a below vertical space were higher than zero. Furthermore, the *D*_irap_ scores of the pro-metaphor stimuli were significantly greater than were those of the anti-metaphor stimuli. Vertical spatial position metaphor of power concepts was verified again by IRAP. These findings suggest that there is an established spatial metaphor for power, which we explain using relational frame theory. It is the first study to our knowledge to explore this metaphor using the IRAP, which overcomes the limitations of paradigms such as the implicit association test, and provides a better understanding of the mechanism of the metaphor.

## Introduction

According to the conceptual processing theory of embodied cognition, most abstract concepts are processed and characterized *via* metaphor. In this way, metaphor acts as a bridge between abstract processing and embodied cognition. The use of metaphor allows individuals, through their own individual and cultural experiences, to form an understanding of and begin processing complex and abstract concepts through simpler, more tangible concepts (Varela et al., 1999; Wilson, 2002).

Power is one such abstract concept that is often constructed through metaphor. Defined as the ability to promote the achievement of one’s own goals through controlling valuable resources (Keltner et al., 2003), power can be metaphorically characterized by weight, color, and size. For example, Lee and Schnall (2014) suggested that power level can influence participants’ judgment of weight: individuals with low power were more likely to judge a box as heavier than were those with high power. Yang et al. (2015) found that power can be metaphorically represented by size and color. In the context of Chinese culture, people often metaphorize something powerful with a large size and the color gold, whereas they metaphorize something powerless with a small size and the color gray.

Previous research has found that abstract concepts such as morality and power are associated with vertical space. Ścigała and Indurkhya (2016) asked subjects to judge morally ambivalent behavior descriptions at the top and bottom of a piece of paper and found that, compared with the descriptions at the bottom of the page, the subjects considered the descriptions at the top of the page to be more moral. Power can also be represented, in both Chinese and English, with spatial metaphors – that is, the use of spatial properties to construct and understand non-spatial concepts (Yin et al., 2013). Vertical position and size are used particularly often in constructing spatial metaphors for power. For example, the phrases 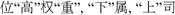 are used by Chinese people to denote that one should “look up to” the “upper class” and “look down upon” the “lower class.” Besides language, it is easy to find examples of spatial metaphors for power in daily life, such as in architecture and social behaviors (e.g., tournament podiums, corporate organizational charts). Schubert (2005) was arguably the first to explore the mechanism of the metaphorical representation of power in vertical orientation. Using a spatial Stroop paradigm, he found that when the power word was consistent with its spatial position (i.e., its perceived power level was consistent with its position in vertical space), the participants tended to discriminate words of high power faster and the level of power as greater. Gagnon et al. (2011) later used the implicit association test (IAT) to explore the connection between verticality and power. They used terrain to associate and represent vertical positions and found that participants were faster at linking mountains with higher power words.

However, some researchers have questioned the repeatability of studies on individual cognition and thus have recommended innovating the experimental paradigm of the study of power (Liu and Liao, 2018). Currently, the paradigms for examining the spatial metaphor of power can be grouped into two categories. The first category consists of paradigms based on group average response times (e.g., Stroop paradigm), wherein power-related vocabulary is positioned in upper or lower vertical positions on a screen and researchers record participants’ average response latency for judging the power level of those words. The strength of the spatial metaphor is considered as the difference in average response latency between pro-metaphor words (i.e., powerful words positioned in the upper vertical space and powerless words in the lower vertical space) and anti-metaphor words (powerful words in the lower vertical space and powerless words in the upper vertical space). However, in relying on the average response latency of groups, these paradigms can merely indicate the existence of spatial metaphor; they are not useful in examining the individual characteristics of metaphor. The other category of paradigm involves use of the IAT, which is based on implicit social cognition. IATs typically use height-related pictures or text (e.g., terrain) to associate and represent vertical positions, which are then combined with power vocabulary for classification. The sensitivity of IATs in evaluating differences has been found to be approximately twice that of the priming paradigm (Greenwald et al., 1998). However, IATs can provide only relative relationship evaluations, and cannot directly assess the essence or directionality of these relationships. In other words, IATs cannot evaluate complex structures with directional relationships, such as spatial metaphor for power (Barnes-Holmes et al., 2006). In addition, in IATs, participants are prone to give false responses, even when asked not to. Similar behavior has been found in Stroop experiments (Kuhl and Kazén, 1999; Kim, 2003; Fiedler and Bluemke, 2005).

A viable alternative to these paradigms is the implicit relational assessment procedure (IRAP), an implicit cognitive measurement paradigm based on relational frame theory (RFT). RFT is based on post-Skinnerian behaviorism and holds that humans recognize and understand the world through *relational responding*. The term “frame” in RFT is an action that refers to “framing events relationally.” This action is the concept of relational responding. In RFT, human beings are recognized and understood through relational responding. When the relationship reaction depends on the context and not simply the physical property of the stimulus, it is a *relational frame*, and the response is known as *arbitrarily applicable relational responding*. The relational frame has three defining features that are used to describe and explain response patterns that emerge in the absence of direct or explicit learning histories (Hughes et al., 2016a). They are (a) *mutual entailment*: if the relationship between A and B is learned, the relationship between B and A can be derived from this, which describes the basic bidirectionality of the relationship reaction; (b) *combinatorial entailment*: the relationship between a new stimulus is determined by deriving the relationship (training or derivation) between two or more stimuli. In a given situation, if A is related to B and A is related to C, then B and C are related in this context; or (c) *transformation of stimulus function*: the stimulation function is based on the relationship of other stimuli to modify the function of a given stimulus, which results in the derived new stimulus having a psychological meaning. When you learn an opposing relationship between A and B through training, and give A the function of “punish” for a certain condition (such as through loss of money), at this time, without direct training or learning, you can obtain B’s “reward” function. There are many types of relational frames, including coordination, opposition, comparative, spatial, and deictic frames. Based on the three definitions above, various relational frames can be combined into relational networks, and that network can then be associated with other relational networks. According to RFT, the associations in relational networks form the basis of psychological processes such as analogy and metaphor (Barnes-Holmes et al., 2001).

**FIGURE 1:**
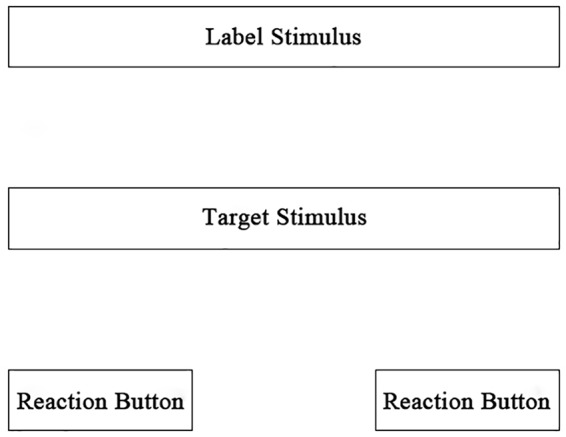
An IRAP stimuli-presenting mode.

Implicit relational assessment procedure is a measurement that was designed to assess the strength or probability of relational responding; it is established during the pre-experimental history of the participant (Hussey et al., 2015), and it is a computer-based task that requires participants to respond quickly and accurately (under time pressure) to sets of stimuli employing a response pattern that may be considered consistent or inconsistent with their previous learning histories (Barnes-Holmes et al., 2010a). In IRAP, each trial contains a target stimulus, a label stimulus, and reaction buttons, providing contextual cues for the relational reaction, usually arranged in the manner shown in Figure 1. The target stimuli are similar to the concept words in IAT (e.g., flowers, insects, or black/white faces), and label stimuli are similar to similar attributive words in IAT (e.g., happy, disgusting), but they are not fixed. A target stimulus can be combined with a label stimulus to form phrases or sentences, for example, “prefer” or “hate” (label stimulus) and “Irish” or “Scots” (target stimulus) (Power et al., 2009); based on the understanding of the spatial relational frame, a label stimulus can provide spatial location by placing the target stimulus in different orientations. Reaction buttons are involved in IRAP, presenting specific relational terms to assess the relationship between related stimuli. The terms’ content is usually consistent/inconsistent, true/false, or similar/opposite. The basic hypothesis is that relational responses should be faster and more accurate on history-consistent than history-inconsistent blocks of trials (Leech et al., 2016). The IRAP directly assesses the relation between stimuli and an individual’s internal relational frame, making it difficult to give false responses. In fact, studies have shown that participants are unable to spontaneously generate a strategy to change their performance on the IRAP – they were only able to provide false responses after being given detailed instructions repetitively throughout the task. In this way, the direction and magnitude of the IRAP effect is more difficult to influence when compared to other implicit tests (Mckenna et al., 2007; Hughes et al., 2016b). The IRAP is also a non-relative measurement method. For example, when discussing the preferences of vegetarians and meat eaters for vegetables and meat, the IAT can only obtain information on vegetarians’ preference for vegetables over meat. By contrast, IRAP can determine the relationship between vegetarians’ preferences for vegetables and meat (Barnes-Holmes et al., 2010b).

Therefore, we used the IRAP to measure the correspondence between powerful and powerless words and upper and lower vertical spatial positions in order to better understand the vertical spatial metaphor of power. Through this method, we expect to analyze the relationship between pro-metaphor and anti-metaphor conditions.

## Materials and Methods

### Participants

Fifty-six undergraduate students (ranging in age from 18 to 22 years old) who volunteered to participate were selected for this study. Thirteen of these students were ultimately excluded because they did not achieve the IRAP performance criteria detailed in the section Instruments and Procedures. All participants had vision that was normal or corrected-to-normal, and their native language was Chinese. Upon completing the experiment, participants received chocolate as a reward.

### Experimental Materials

Twelve words were selected from Schubert’s (2005) and Zanolie et al.’s (2012) experimental materials, including six powerful words (principal, academician, government, boss, professor, policeman) and six powerless words (slave, servant, prisoner, security, student, worker). Before the experiment, 19 college students who did not participate in the formal experiment were recruited to judge these 12 words along two dimensions: power level and familiarity. They used a scale ranging from 1 (powerful/familiar) to 7 (powerless/unfamiliar). The results indicated that the powerful words (*M* = 2.40, *SD* = 0.65) and the powerless words (*M* = 5.79, *SD* = 0.45) differed significantly in their power level scores [*F*(1,17) = 538.54, *p* < 0.001]. The words did not, however, differ significantly in their familiarity; in all cases, participants rated the words as less than 3 [*F*(1,17) = 0.01, *p* = 0.946].

### Instruments and Procedures

**FIGURE 2:**
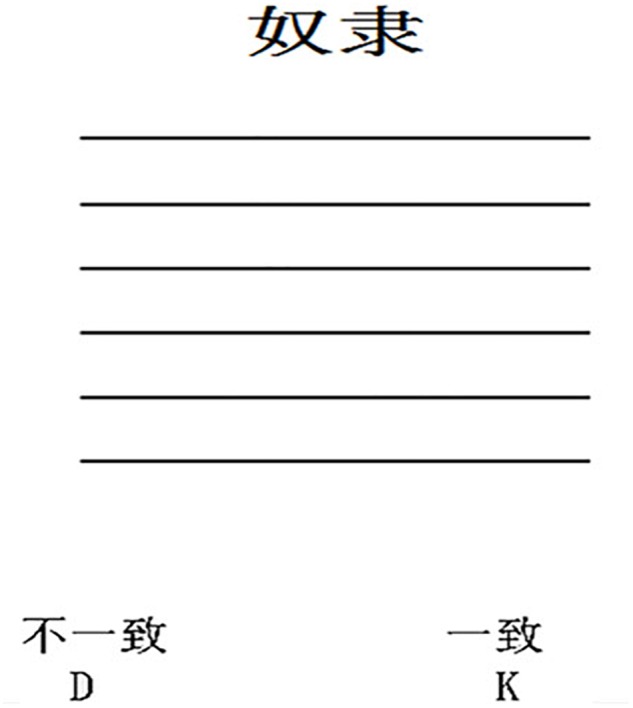
An example of experimental presentation material.

The experiment was run in E-prime 2.0, which also collected the experimental data. The IRAP we used comprised two practice blocks and a fixed set of six test blocks (Barnes-Holmes et al., 2006). Each block had the same number of trials and comprised four different trial types denoting different stimulus relationships. The four trial types were defined in terms of their power level (powerful or powerless words) and the position of the stimuli on the computer screen along the vertical axis (top or bottom), using the following two labels: (1) “pro-metaphor,” in which a powerful word was presented at the top of the computer screen (powerful-high) or a powerless word was presented at the bottom of the screen (powerless-low); and (2) “anti-metaphor,” where the powerful word was presented at the bottom of the computer screen (powerful-low) or the powerless word at the top of the screen (powerless-high). In each block, each target word was once presented for each of the two labels (for a total of 24 trials per block). When the vocabulary was presented, six equally spaced straight lines appeared above or below each word. Furthermore, “consistent” and “inconsistent” answer options were displayed on the left and right sides of the screen (the relative positions of these options was randomized) (see Figure 2).

Each block presented one of two types of task: compatible or incompatible. The compatible task was designated as instructing participants to judge the pro-metaphor stimulus as “consistent” and the anti-metaphor stimulus as “inconsistent”; otherwise, it was considered an incompatible task. The tasks were alternated between blocks. To balance the sequential effects, we randomly divided participants into initial compatibility and initial incompatibility conditions.

Before each block, participants were asked to carefully read the instructions and then to make judgments about the consistency of the word and its vertical spatial position according to those instructions. They were to respond by pressing a button: If the answer was on the left, they were to press the “D” key, whereas they pressed the “K” key if the answer was on the right.

In the practice blocks, the participants were informed that they were participating in a practice phase and that errors are expected. When participants responded correctly in a trial, the screen was cleared for 400 ms, after which the next trial was presented; by contrast, when they responded incorrectly, a red “x” appeared at the bottom of the screen, and the next trial did not begin until the participant pressed the correct response key. On-screen feedback detailing the percentage of correct responses and the median response latency for the block was provided after each block. If the correct answer rate did not reach 80% or the median response latency was more than 3,000 ms, they were asked to continue practicing. If they failed to meet these criteria after three attempts, they were thanked for their participation and their data were discarded. Participants who met the criteria entered the formal test phase. These criteria were used to ensure that participants understood and followed the instructions of the IRAP (Barnes-Holmes et al., 2010a).

The test blocks were similar to the practice blocks, except that the instructions reminded participants to complete the trials as quickly and as accurately as possible. We discarded all data for a participant who had any block with a correct answer rate of less than 80% or a median response latency of more than 3,000 ms.

### Data Processing

Referring to Barnes-Holmes et al. (2010a) and Greenwald et al. (2003), we calculated *D*-scores (*D*_irap_) for the four types of stimulus relationship to be used as the dependent variables. The *D*_irap_ was derived from the response latencies of compatible and incompatible tasks. *D*-scores can effectively minimize the effects of age, motor skills, and cognitive abilities on response latency, as well as reduce the impact of extraneous variables associated with individuals. The data were analyzed using R.

The steps involved in calculating the *D*_irap_ scores are as follows:

(1) Only response-latency data from test blocks are used;
(2) Latencies above 10,000 ms from the data set are eliminated;
(3) All data for a participant are removed if he or she produces more than 10% of test-block trials with latencies less than 300 ms;
(4) Twelve standard deviations for the four trial types are computed: four from the response latencies from Test Blocks 1 and 2, four from the latencies from Test Blocks 3 and 4, and a further four from Test Blocks 5 and 6;
(5) Twenty-four mean latencies for the four trial types in each test block are calculated;
(6) Difference scores are calculated for each of the four trial types for each pair of test blocks by subtracting the mean latency of the compatible block from the mean latency of the corresponding incompatible block;
(7) Each difference score is divided by its corresponding standard deviation from step 4, yielding 12 *D*_irap_ scores, one score for each trial type for each pair of test blocks;
(8) Four overall trial-type *D*_irap_ scores, or IRAP effects, are calculated by averaging the scores for each trial type across the three pairs of test blocks.

## Results

### Mean *D*_irap_ Scores

**FIGURE 3:**
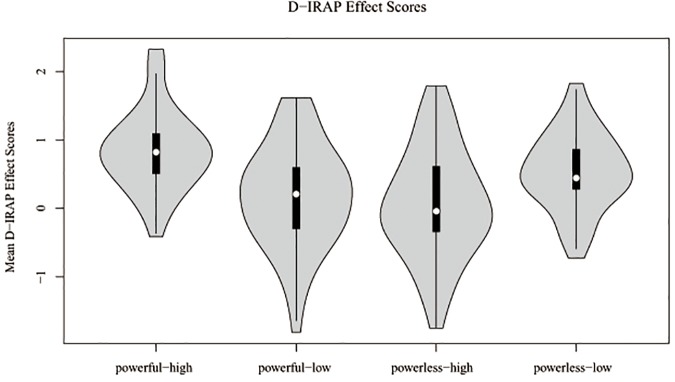
Mean *D*_irap_ effect scores.

The mean *D*_irap_ scores of the four types of stimulus relationship are shown in Figure 3. For the pro-metaphor stimuli (powerful-high and powerless-low), the *D*_irap_ score was high, whereas they were rather low for the anti-metaphor stimuli (powerful-low and powerless-high words).

A one-sample *t* test was then performed to examine whether the *D*_irap_ scores of the four types of stimulus relationship were significantly different from zero. We found that the *D*_irap_ score of powerful-high stimuli was 0.84, *t*(45) = 9.78, *p* < 0.0001, Cohen’s *d* = 1.44, 95% confidence interval [CI] = [0.67, 1.02]; that of powerful-low stimuli was 0.17, *t*(45) = 1.61, *p* = 0.114, Cohen’s *d* = 0.23, 95% CI = [-0.04, 0.40]; that of powerless-high stimuli was 0.06, *t*(45) = 0.49, *p* = 0.625, Cohen’s *d* = 0.07, 95% CI = [-0.18, 0.29]; and that of powerless-low stimuli was 0.54, *t*(45) = 6.53, *p* < 0.0001, Cohen’s *d* = 0.96, 95% CI = [0.37, 0.71]. These findings indicated that for the pro-metaphor stimuli, there was a significant relationship between power vocabulary and vertical space is significant, whereas no such association was found for the anti-metaphor stimuli.

### Differences in *D*_irap_ Scores by Stimulus Relationship

Next, we entered *D*_irap_ scores into the one-way repeated-measures ANOVA with trial type (i.e., stimulus relationship) as the independent variable. The main effect of trial type was significant, *F*(3,45) = 15.937, *p* < 0.0001, η_p_^2^ = 0.262; a *post hoc* test (least significant difference) revealed that the *D*_irap_ of the pro-metaphor stimuli was significantly higher than that of the anti-metaphor stimuli (*p* values < 0.01), as follows: powerful-high versus powerful-low, *p* < 0.001; powerful-high versus powerless-high, *p* < 0.001; powerless-low versus powerful-low, *p* = 0.003; and powerless-low versus powerless-high, *p* = 0.001. There is also a significant difference between powerful-high and powerless-low (*p* = 0.001) while comparison with powerful-low and powerless-high was not significant (*p* = 0.302).

### Split-Half Reliability

To assess the internal consistency of the IRAP, four split-half reliability scores were calculated. In each case, two scores (one for odd trials and one for even trials) were calculated, and these were obtained in the same way as for the four original scores, except that the algorithm described previously was applied separately to all odd trials and to all even trials. The results were as follows: powerful-high, *r* = 0.78, *p* < 0.001; powerful-low, *r* = 0.87, *p* < 0.001; powerless-high, *r* = 0.84, *p* < 0.001; powerless-low, *r* = 0.94, *p* < 0.001.

## Discussion

The IRAP was used to explore the vertical space metaphor of power in this study. We found that the *D*_irap_ score was significantly different from zero when powerful words were placed in the upper vertical position and powerless words were positioned in the lower position. Furthermore, the *D*_irap_ scores of pro-metaphor stimuli were significantly greater than those of anti-metaphor stimuli, and there is also a significant difference between powerful-high and powerless-low.

According to the relationship elaboration and coherence (REC) model proposed by Barnes-Holmes (2010), in the IRAP, initial relational responding – that is, the act of constructing events in a relational manner, which is largely determined by individuals’ experience and current context – is rapidly completed before participants actually press the response key. The rapidity of their response suggests that the response is the strongest relation in their mind (e.g., the spatial relational frame of power). In our experiment, when these initial relational responses were consistent with the experimental requirements (also known as the compatible task, e.g., pressing the key corresponding to “consistent” when the powerful word is in the upper vertical space), participants tended to react more quickly; otherwise (i.e., in the incompatible task), participants needed to correct their initial relational responding to fit the task, leading to an increase in their reaction time. In sum, since the incompatible task requires modification of a psychological process, reaction times should be longer when compared to those in the compatible task; thus, the difference between the two tasks is the strength of the specific belief (i.e., spatial metaphor) assessed by the IRAP. Our findings accord with those of previous studies conducted in China, where powerful words corresponded with an upper vertical position and powerless words corresponded with a lower position, while also building on these studies by using the IRAP (Tang et al., 2015).

**FIGURE 4:**
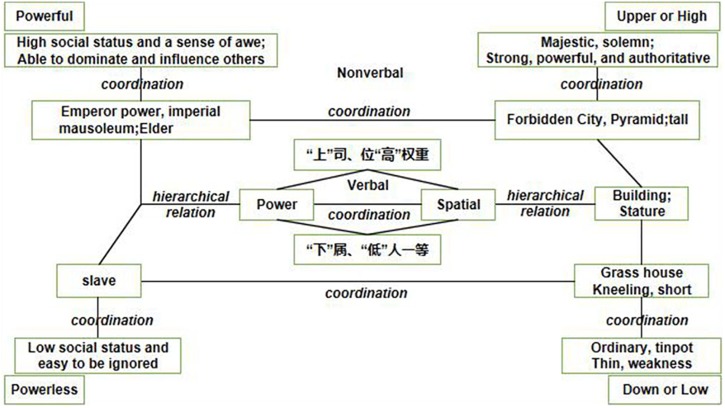
An RFT analysis of the vertical spatial metaphor of power.

Thus, how is spatial metaphor formed? RFT explains the formation of analogy and metaphor from the perspective of behaviorism. Specifically, this theory suggests that analogy is achieved through mutual and combinatorial entailment of relational networks. Metaphor, then, is based on consolidation of the common features of two obviously different events achieved by transformation of the stimulus function (Barnes-Holmes et al., 2001). Figure 4 shows the translation of the spatial metaphor of the power into the language of RFT (Foody et al., 2014). In the present experiment, *D*_irap_ scores for metaphorically inconsistent stimuli did not significantly differ from zero and were significantly lower than the scores of metaphorically consistent stimuli. These findings support RFT’s explanation of the formation of spatial metaphors. More specifically, in the compatible tasks for anti-metaphor stimuli, participants were required to judge powerful-low and powerful-high words as “inconsistent,” which aligns with people’s overt perceptions. However, these stimulus relationships do not reflect their implicit attitudes. This can be explained by the fact that certain implicit attitudes remain stable throughout life, corresponding with “grounded experience” in weak embodied cognition, which implies that cognitive phenomena such as memory, cognition, reasoning, social ranking, and even moral values are mostly mediated and shaped by our everyday life experiences (Tirado et al., 2018). In the case of the vertical spatial metaphor of power, this might have to be due to its ubiquity: in Chinese culture, verbal expressions are often used to denote this spatial metaphor, such as 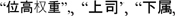 and 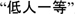. Architecture has also been used to highlight power through spatial elements – the height of the Forbidden City was, for instance, used to emphasize the power of the emperor. Chinese culture also has a strict emphasis on obedience and awe of power status; kneeling is used as a gesture of respect for others, as it denotes them to be of higher social status (Li et al., 2016). Foreign studies have shown that the vertical spatial metaphor of power has cross-cultural consistency. This is evident simply by looking at skyscrapers, pyramids, and cathedrals, all of which are used to symbolize power (Cian, 2017). In the family domain, taller brothers and sisters and other children taller than themselves can occupy the dominant position in the group through physical strength (Schwartz et al., 1982). This close relationship between height and power is formed throughout individuals’ development, which might lead to a ubiquitous bias toward perceiving height as representative of social power. Even after adulthood, this bias is difficult to change. Indeed, taller people tend to have higher average wages than do shorter ones (Gagnon et al., 2011). Leadership positions are often occupied by tall people, leading them to have higher social status and political power (Judge and Cable, 2004). Politicians are also considered to be in a higher position after winning an election (Higham and Carment, 1992). In marketing, when the brand identity is positioned relatively high on packaging, consumers tend to prefer more powerful brands (e.g., Apple), whereas when the brand identity is in a relatively low position on the packaging, they prefer more powerless brands such as Gateway (Sundar and Noseworthy, 2014). All of these phenomena suggest that when children try to understand the abstract concept of power, they use space as a metaphor – they take note of high-rise buildings that give people a sense of majesty and inviolability, and of the perceived strength and power of tall people. These meanings are given to power *via* the transformation of stimulus function, which enables children to understand the concept of power.

There are still some theories that explain spatial metaphors, such as Perceptual Symbols Theory, Simulating Sensorimotor Metaphors, and Conceptual Metaphor Theory, among which Structure Mapping Theory is also an interesting theory to describe metaphor mapping. Structure mapping theory describes how to understand abstract concepts by aligning and adjusting the characteristics of concrete concepts, so that these concepts can become a “working template” (Marmolejo-Ramos et al., 2018). Knowing these theories is comprehensive to understand spatial metaphors.

This study discusses the spatial metaphors of power. In the Chinese context, left and right, size and italics have certain meanings, such as 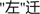 and 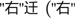 means “left,” 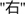 means “right”; 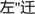 means demotion, 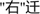 means promotion). In the future, we can try to use IRAP to explore the spatial metaphors of other attributes, such as left and right of power.

## Data Availability

The raw data supporting the conclusions of this manuscript will be made available by the authors, without undue reservation, to any qualified researcher.

## Ethics Statement

This study was carried out in accordance with the recommendations of Ethical Principles of Psychologists and Code of Conduct from the American Psychological Association with written informed consent for all participants. Moreover, the experiment was a computer button task and the stimuli used were names of general jobs; the risk to the participants would not be greater than that in daily life.

## Author Contributions

BH participated in the design, data collection, data analysis, data interpretation, and drafting the early version of the article. LZ participated in the design, data collection, and drafting the early version of the article. HS participated in the design and revising the article critically for better intrinsic logicality.

## Conflict of Interest Statement

The authors declare that the research was conducted in the absence of any commercial or financial relationships that could be construed as a potential conflict of interest.
